# Genome-Wide Identification, Phylogenetic Analysis and Salt-Responsive Expression Profiling of the MYB Transcription Factor Family in *Cannabis sativa* L. During Seed Germination

**DOI:** 10.3390/ijms27021087

**Published:** 2026-01-22

**Authors:** Di Wang, Shuyue Liang, Ye Che, Guochao Qi, Zeyu Jiang, Wei Yang, Haohan Zhao, Jikang Chen, Aiguo Zhu, Gang Gao

**Affiliations:** 1 Daqing Branch of Heilongjiang Academy of Agricultural Sciences, Daqing 163319, China; dqnkywd@126.com (D.W.);; 2 Institute of Bast Fiber Crops, Chinese Academy of Agricultural Sciences, Changsha 410205, China; 3 Yuelushan Laboratory, Changsha 410128, China

**Keywords:** *Cannabis sativa*, MYB transcription factor, genome-wide analysis, salt stress, seed germination, gene expression

## Abstract

Seed germination is a critical developmental stage exhibiting high vulnerability to salt stress. The role of MYB transcription factors (TFs) in mediating this process in *Cannabis sativa* L. remains largely unexplored. In this study, we performed a genome-wide analysis and identified 115 *CsMYB* genes, which were phylogenetically classified into 12 distinct subgroups. In silico promoter analysis revealed a significant enrichment of abscisic acid (ABA)- and methyl jasmonate (MeJA)-responsive cis-elements, suggesting their potential linkage to phytohormone signaling pathways under stress conditions. To investigate their expression during salt stress, we profiled a subset of candidate *CsMYB* genes during seed germination under 150 mM NaCl treatment based on RNA-seq screening at 24 h post-imbibition (hpi) under salt stress. These candidates exhibited distinct temporal expression profiles: *CsMYB33* and *CsMYB44* were transiently induced at the early stage (12 h post-imbibition), while *CsMYB14*, *CsMYB78*, and *CsMYB79* showed sustained upregulation from 24 h to 5 days. In contrast, *CsMYB58* and *CsMYB110* were downregulated. Synteny analysis indicated a closer evolutionary relationship between *CsMYBs* and their *Arabidopsis thaliana* orthologs compared to those in monocots. Protein–protein interaction predictions, based on orthology, further implicated these *CsMYBs* within putative ABA signaling and reactive oxygen species (ROS) homeostasis networks. Collectively, our findings provide a systematic genomic identification and genomic characterization of the *CsMYB* family and propose a model for the potential multi-phase involvement of selected CsMYBs in the salt stress response during seed germination. This work establishes a foundational resource and identifies key candidate genes for future functional validation aimed at enhancing salt tolerance in *C. sativa*.

## 1. Introduction

Environmental stresses, particularly soil salinity, constitute significant constraints on global crop productivity by disrupting plant growth and development [1,2]. Seed germination represents a critical and particularly susceptible phase in the plant life cycle, during which salinity imposes both osmotic and ionic stresses, resulting in delayed germination, reduced germination rates, and impaired seedling establishment [3,4]. Elucidating the molecular mechanisms underpinning tolerance during this pivotal stage is therefore essential for breeding resilient crops.

Transcription factors (TFs) function as master regulators orchestrating complex transcriptional reprogramming in response to abiotic stresses [5,6]. Among these, the MYB (v-myb avian myeloblastosis viral oncogene homolog) family constitutes one of the largest and most functionally diverse TF families in plants. MYB proteins are characterized by a conserved DNA-binding domain and are extensively implicated in processes ranging from secondary metabolism and development to phytohormone signaling and stress adaptation [7,8]. Notably, several MYB TFs have been identified as key mediators of salt stress responses. For instance, *Arabidopsis* AtMYB44 enhances abiotic stress tolerance by modulating stomatal aperture and reactive oxygen species (ROS) homeostasis [9], while overexpression of wheat TaMYB33 improves salinity and drought resilience in transgenic lines [10]. These findings underscore the potential of MYB TFs as targets for the genetic enhancement of stress tolerance.

*Cannabis sativa* L. is a multipurpose crop of increasing agronomic and economic significance, valued for its fibers, seeds, and a rich spectrum of bioactive secondary metabolites, including cannabinoids and terpenes [11,12,13]. Globally, industrial hemp (a low-THC variety of *C. sativa* is cultivated for fiber, seed, and phytochemical production, with an estimated market value exceeding USD 5 billion annually [14]. Its seeds are rich in protein and essential fatty acids, while its fibers are used in textiles, construction, and biocomposites, contributing to a sustainable bioeconomy. Furthermore, cannabinoids such as cannabidiol (CBD) have gained prominence in the pharmaceutical and wellness industries, driving rapid expansion in licensed cultivation. However, the productivity and metabolic consistency of *C. sativa* are highly vulnerable to abiotic stresses. Among these, soil salinity represents a pervasive and escalating constraint to global agriculture, affecting over 20% of irrigated land worldwide. The cultivation of hemp on marginal or salt-affected soils is increasingly considered as a strategy for sustainable land use, yet its successful implementation is hampered by the species’ sensitivity to salt, particularly during the critical stage of seed germination [15,16].

Seed germination is arguably the most vulnerable phase in the plant life cycle, determining successful crop establishment. Under salinity, germinating seeds face immediate osmotic stress that restricts water uptake, followed by ionic and oxidative stresses that disrupt cellular homeostasis. This leads to delayed or arrested germination, reduced seedling vigor, and ultimately crop failure. Therefore, elucidating the molecular mechanisms that confer salt tolerance during this pivotal window is essential for breeding resilient hemp varieties capable of establishing in saline environments. Although MYB transcription factors are known to participate in diverse abiotic stress responses (e.g., drought, cold, and heavy metals), their specific roles and regulatory networks during salt-stressed seed germination in *C. sativa* remain entirely unexplored.

To address this gap, we performed the genome-wide identification and characterization of the MYB TF family in *C. sativa*. We identified 115 *CsMYB* genes and conducted phylogenetic, structural, and promoter analyses. Furthermore, by profiling expression dynamics during salt-stressed germination, we identified members with distinct temporal expression patterns. Integrated with in silico promoter and protein interaction analyses, these data suggest that specific CsMYBs are associated with early and sustained phases of the salt stress response and are potentially connected to ABA and ROS-related pathways. This study provides a foundational genomic resource and set of high-priority candidate genes for the future experimental validation of MYB function in improving salinity tolerance during the critical germination stage of this economically vital crop.

## 2. Results

### 2.1. Genome-Wide Identification and Characterization of MYB Genes in C. sativa

To comprehensively elucidate the MYB transcription factor family in *C. sativa*, a genome-wide identification analysis was conducted. A total of 115 non-redundant *CsMYB* genes were identified, confirming the MYB family as a major transcription factor family in *C. sativa*. The physicochemical properties of deduced *CsMYB* proteins were analyzed using ProtParam (https://www.expasy.org/). Protein length exhibited considerable variation, ranging from 202 amino acids (*CsMYB63*) to 1262 amino acids (*CsMYB1*). Correspondingly, molecular weights (MW) varied widely from approximately 23.40 kDa to 142.45 kDa, with a mean MW of 45.21 kDa. Theoretical isoelectric points (pI), indicating the pH at which proteins carry no net charge, spanned a broad spectrum from 4.71 (*CsMYB104*) to 9.63 (*CsMYB45*), with an average pI of 6.79. This pI diversity suggests functional diversification, as isoelectric points influence protein solubility, subcellular localization, and interaction partners [17].

Chromosomal localization analysis revealed an uneven distribution of the 115 *CsMYB* genes across all 10 chromosomes of *C. sativa*. Notably, certain chromosomes harbored significantly more *CsMYB* genes (e.g., Chromosomes 1 and 5), while several genes formed tandem clusters. This pattern suggests gene duplication events, particularly tandem duplications, as a major driver of *MYB* gene family expansion in *C. sativa* [18].

### 2.2. Phylogenetic Classification and Motif Analysis

**Figure 1:**
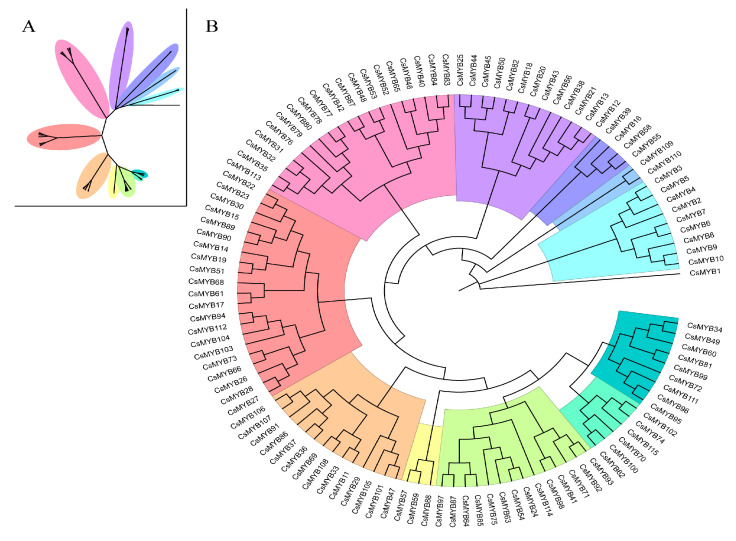
Phylogenetic analysis of MYB transcription factors in *C. sativa*. (**A**) Unrooted phylogenetic tree. (**B**) Rooted proportional tree. The phylogenetic tree was constructed using the Maximum Likelihood method in MEGA 12 software [19].

**Figure 2:**
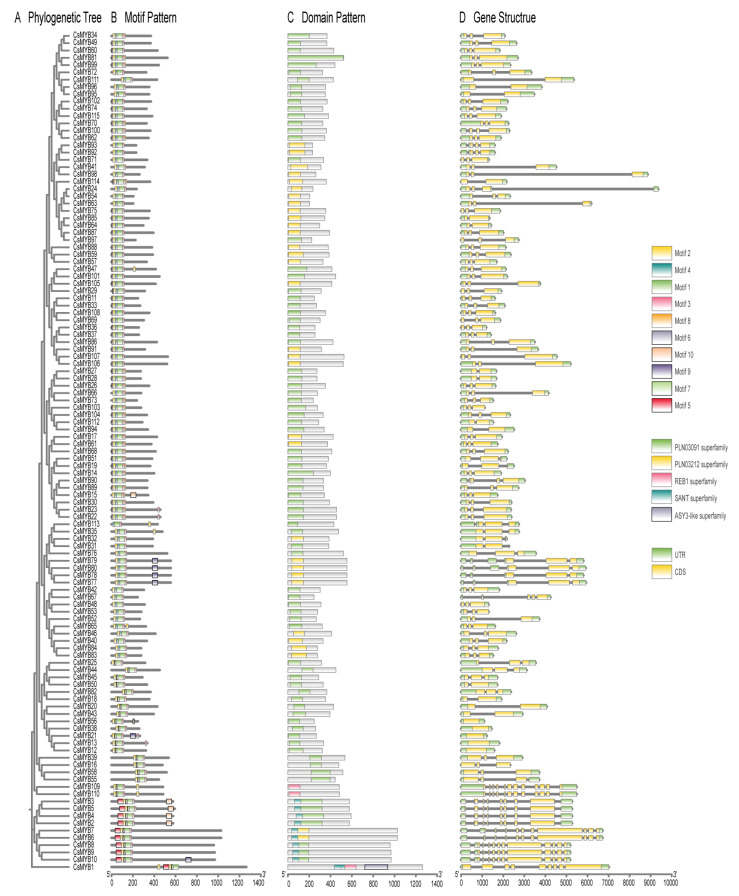
(**A**). Phylogenetic analysis tree (constructed using MEGA 12 [19]). (**B**). Motif pattern of *CsMYB* genes. 10 different motifs are displayed in different colored boxes. (**C**). Analysis of conserved domains. (**D**). Exon-intron structure of *CsMYB* genes. Yellow boxes indicate exons; green boxes indicate untranslated 5′- and 3′-regions; black lines indicate introns.

The phylogenetic tree revealed that *CsMYB* genes could be classified into 12 subgroups (Figure 1), consistent with those in *A. thaliana* [19]. Motif analysis showed that members within the same subgroup shared similar motif compositions (Figure 2B), indicating functional conservation. Ten conserved motifs were identified and the number of motifs in the *CsMYB* proteins ranged from a minimum of four to a maximum of seven. Among all *CsMYB* proteins, only *CsMYB22* and *CsMYB23* contained seven motifs, while most others contained 4–6 motifs. The most prominent motif was motif 2, which was present in all 115 *CsMYB* proteins, making it possibly the most critical motif. In contrast, motif 10 was the least common and was found in *CsMYB2*, *CsMYB3*, *CsMYB4*, *CsMYB5* and *CsMYB15*. The functional diversity of MYB gene family may be related to the diversity of motif distribution [20].

### 2.3. Gene Structure and Promoter Analysis

*CsMYB* genes exhibited varied exon-intron structures, with introns ranging from 1 to 11 (Figure 2D). Most *CsMYB* genes contained 2–3 introns, while *CsMYB109* and *CsMYB110* contained the highest number of introns (11). There was a certain similarity in the number and distribution of exon-introns within the same subgroup, for example, *CsMYB109* and *CsMYB110* are assembled together in the phylogeny tree, and both of them contained eleven introns with the same distribution patterns. In order to explore the potential functions of *CsMYBs*, a prediction was made for the cis-element located 2000 bp upstream of the transcription start site of *CsMYBs.*

**Figure 3:**
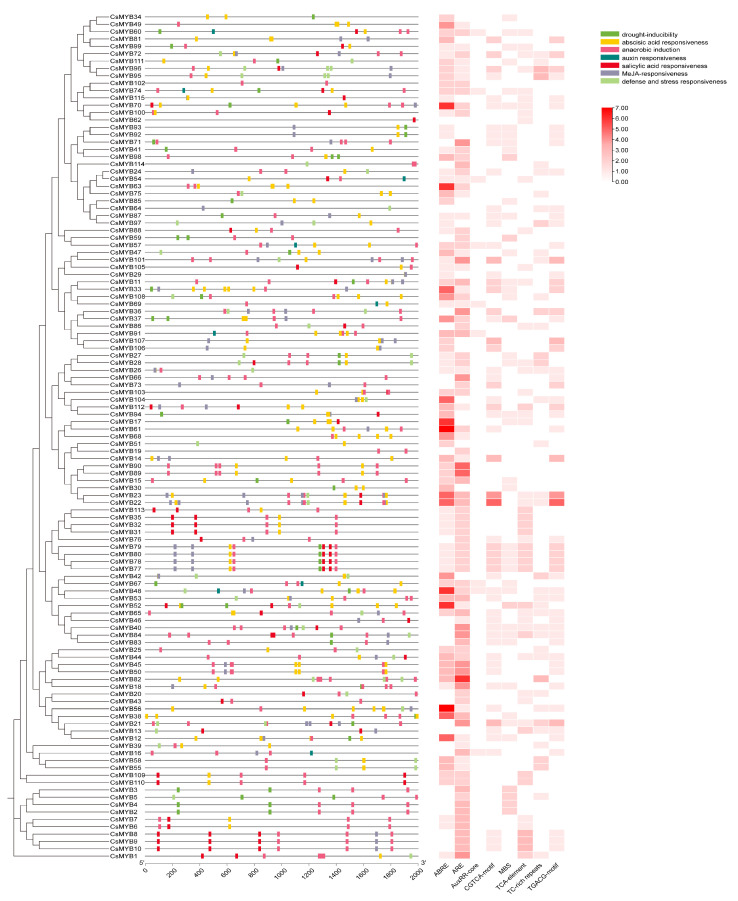
Cis-elements promoter region (2000 bp upstream) of *CsMYB* genes with a heat map displaying stress-responsive elements and indicated by seven colors. The number of the rectangular boxes represents the number of cis-elements. Cis-elements were predicted using the PlantCARE database [21,22].

Promoter analysis identified the cis-elements related to ABA, JA, SA, auxin, and stress responses, suggesting roles in hormone signaling and stress adaptation (Figure 3) [21]. By screening and classifying an abundant number of detected cis elements, a total of 8 cis-elements related to the above were obtained. The cis-elements that respond to plant hormones were mainly methyl jasmonate (MeJA), abscisic acid (ABA), and auxin responsive elements. Among them, ARE elements were the most abundant (220 in total), followed by ABRE (217 in total), CGTCA-motif (85 in total, MeJA responsive) and TGACG-motif (85 in total, MeJA responsive). In terms of stress response, TC-rich repeats (54 in total, stress response) were more abundant, followed by MBS (51 in total, drought response). Additionally, the *CsMYB* genes with similar cis-element types and numbers were shown to have close phylogenetic relationships. For example, *CsMYB8*, *CsMYB9*, and *CsMYB10* all have 3 ARE, 3 TCA-elements, 1 CGTCA motif and 1 TGACG motif, suggesting that the gene subgroup may have potential regulatory connections to ABA, salicylic acid, and methyl jasmonate signaling pathways. These results further reveal the potential function of *CsMYB* genes and their role in environmental stress responses [22].

### 2.4. CsMYBs Chromosome Distribution and Synteny Analysis

**Figure 4:**
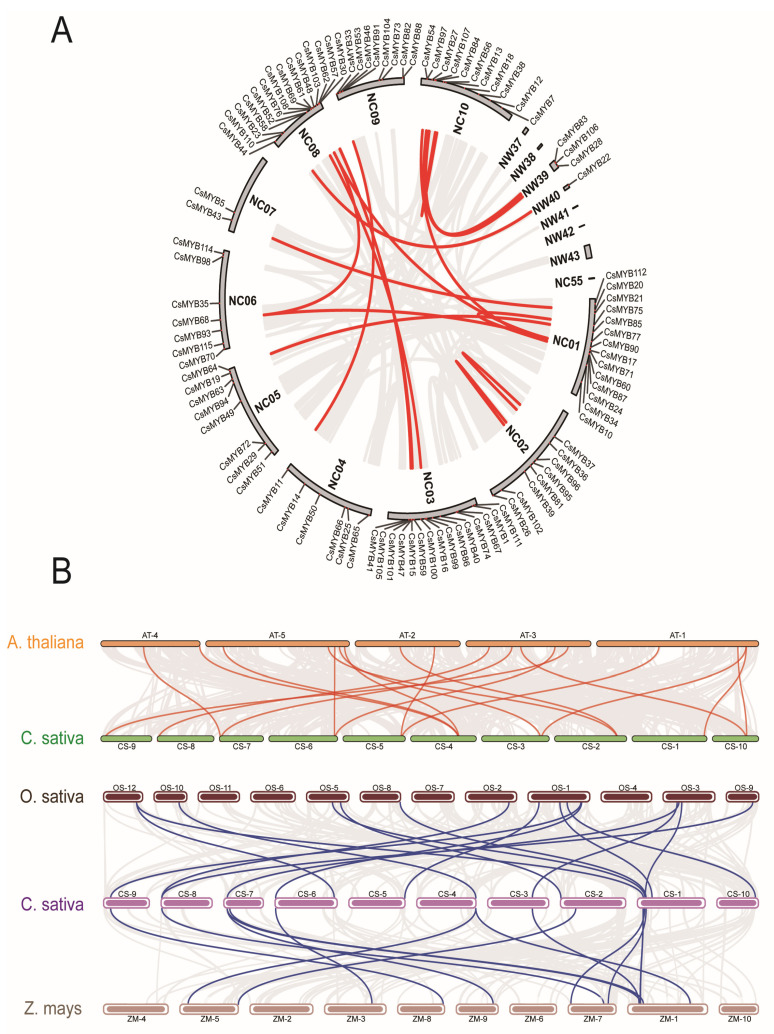
The *C. sativa* MYB gene chromosomal distribution and their relationships (**A**) and Synteny analyses of *C. sativa* MYB genes with *Arabidopsis thaliana* on the one hand and with *Oryza sativa* and *Zea mays* on the other hand (**B**). Synteny analysis was performed using the MCScanX algorithm in TBtoolsv 1.098 [23,24].

The 115 *CsMYB* genes were distributed across 18 chromosomes/scaffolds (NC1–NC10; NC55; NW37-43) in the *C. sativa* genome (Figure 4A). The number of MYB genes on NC03 is the highest (15), followed by NC01 and NC08 (14), and NC10 (11). There is no MYB gene on chromosomes (NW37, NW38, NW41, NW42, NW43, and NC55). The number of *MYB* genes on NW40 is the least (1), followed by NC07 (2) and NW39 (3). NC02, NC05, and NC09 each have 8 genes, with 7 genes on NC06 and 6 genes on NC04. There is no significant correlation between the number of genes on chromosomes and the length of chromosomes. The density of *CsMYB* at the bottom of NC08 and the top of NC09 is relatively high.

The syntenic analysis of the *CsMYBs* and *MYBs* from *A. thaliana*, *O. sativa* and *Z. mays* revealed a markedly higher number of orthologous gene pairs between *C. sativa* and *A. thaliana* (48 pairs) compared to those with *O. sativa* (18 pairs) and *Z. mays* (15 pairs) (Figure 4B). This quantitative disparity supports a closer evolutionary relationship between *C. sativa* and the dicot model *A. thaliana*, consistent with their shared phylogenetic lineage, and underscores the conserved evolution of the MYB gene family within dicots [23]. In contrast, the lower degree of collinearity with the monocot species (*O. sativa* and *Z. mays*) suggests that substantial divergence occurred after the monocot–dicot split, with many MYB genes likely evolving independently in each lineage [24].

### 2.5. Expression Profiles Under Salt Stress and During Germination

#### 2.5.1. RNA-Seq Analysis and Candidate Gene Selection

To obtain an initial set of salt-responsive candidates, a preliminary RNA-seq experiment was performed on seeds germinating under 150 mM NaCl at a single time point of 24 h post-imbibition (hpi). Differential expression analysis identified a subset of *CsMYB* genes that were significantly upregulated or downregulated under salt stress compared to the water control (see Supplementary Table S2). Based on this screening data, combined with the presence of stress/hormone-related cis-elements in their promoters and phylogenetic proximity to known stress-responsive MYBs in *Arabidopsis*, ten candidate *CsMYB* genes (*CsMYB14*, *CsMYB19*, *CsMYB33*, *CsMYB44*, *CsMYB58*, *CsMYB63*, *CsMYB78*, *CsMYB79*, *CsMYB101*, *CsMYB110*) were selected for detailed temporal expression profiling via qRT-PCR.

#### 2.5.2. Temporal Expression Dynamics via qRT-PCR

**Figure 5:**
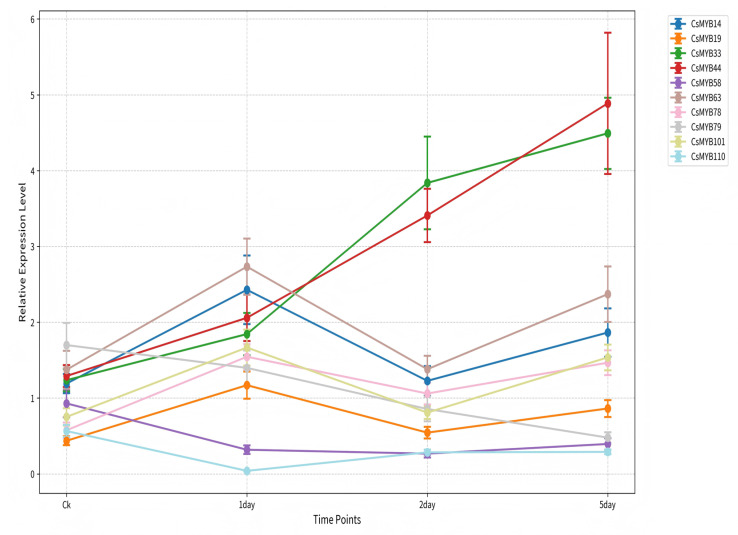
Relative expression of selected *CsMYB* genes during the different germination time of *C. sativa* seeds under salt stress (150 mM).

The expression dynamics of the ten candidate CsMYB genes were analyzed at five time points (0, 12, 24, 48 h, and 5 d) during seed germination under both control (water) and 150 mM NaCl treatment (Figure 5). To clearly distinguish salt-specific responses from normal developmental expression, the expression fold-change (NaCl/Control) is presented.

The results showed that most *CsMYB* genes exhibited significant expression regulation patterns under salt stress (Figure 5) [25]. Among them, *CsMYB14*, *CsMYB78*, and *CsMYB79* showed significantly increased expression levels after 24 h of salt stress treatment, being 4.2 times, 5.6 times, and 6.1 times that of the control group (*p* < 0.05), and maintained high expression levels at 48 h, consistent with a role in the early to mid-phase response to salt stress. It is notable that the expression levels of *CsMYB78* and *CsMYB79* further increased at 5 d, indicating a potential involvement in maintaining responses during the later stages of germination. In contrast, *CsMYB33* and *CsMYB44* showed rapid induction of expression at 12 h of salt stress treatment, but their expression gradually declined after 48 h, showing an early transient response characteristic. Additionally, *CsMYB58* and *CsMYB110* were significantly suppressed under salt stress across multiple time points, suggesting that they may have functions in normal germination, while salt stress may inhibit their expression to adjust metabolic pathways [25,26].

### 2.6. Protein–Protein Interaction and Regulatory Network Analysis of CsMYB

To generate preliminary hypotheses regarding potential functional contexts, a putative protein–protein interaction (PPI) network was inferred. Leveraging the STRING database, interactions were predicted based on orthology transfer from experimentally characterized *A. thaliana* MYB proteins and their known interactors, as direct experimental evidence in *C. sativa* is currently lacking.

**Figure 6:**
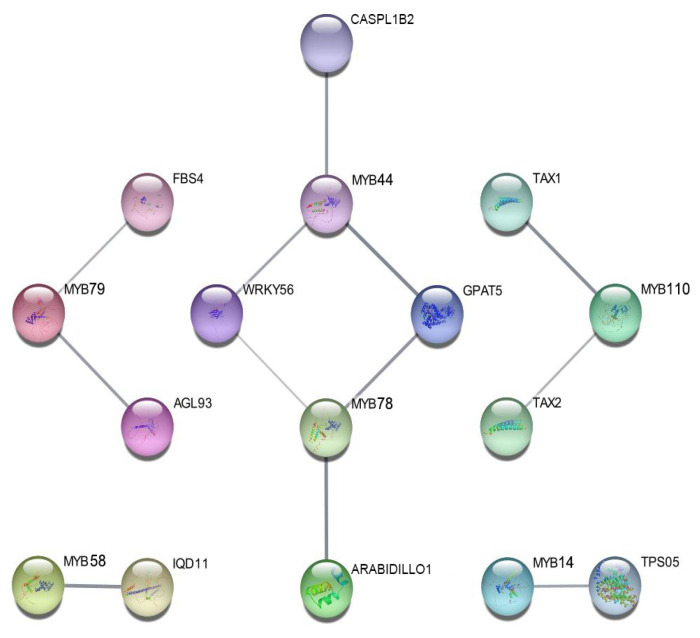
Predicted protein–protein interaction network of CsMYB transcription factors. The network was constructed using the STRING database based on orthology to *A. thaliana* interaction data and visualized with Cytoscape software web 1.0 [27]. Nodes denote proteins (CsMYB proteins and their predicted interaction partners), and edges signify functional associations, with the line thickness corresponding to the confidence score of the interaction. The layout depicts potential functional modules and highlights central nodes involved in the salt stress response.

The resultant predicted network (Figure 6) reveals a complex and interconnected architecture, suggesting putative functional partners for core *CsMYB* members. Notably, several *CsMYBs* identified as salt-responsive in our expression analysis, such as *CsMYB14*, *CsMYB78*, and *CsMYB79*, are predicted to interact with proteins participating in crucial stress-response pathways, including components of ABA signaling (e.g., protein phosphatases 2C and SnRK2 kinases) and enzymes accountable for reactive oxygen species (ROS) homeostasis (e.g., peroxidases and catalases). Moreover, the network implies extensive cross-talk among different *CsMYB* members themselves, as well as with other families of stress -related transcription factors (e.g., b*ZIPs*, *NACs*), suggesting coordinated multi-level transcriptional regulation. For example, *CsMYB44*, which demonstrated an early transient induction pattern is predicted to connect several regulatory modules. This in silico PPI analysis provides a systems-level perspective and testable hypotheses regarding potential cooperative functions of *CsMYBs*, which require future experimental validation in *C. sativa* [27].

## 3. Discussion

Seed germination is a critical phase in the plant life cycle, determining successful crop establishment. This process is highly sensitive to abiotic stresses, with salinity being a major constraint that imposes osmotic and ionic stresses, leading to delayed germination, reduced germination rates, and poor seedling vigor [3,15,28]. Understanding the molecular mechanisms underpinning salt stress tolerance during this vulnerable stage is therefore essential for improving crop resilience. This study provides the comprehensive genome-wide analysis of the MYB transcription factor family in *C. sativa* L. and delineates its putative multi-layered regulatory roles during salt-stressed seed germination. Our integrated findings reveal distinct temporal patterns of *CsMYB* expression under salt stress, which align with conserved stress-response frameworks in plants while also highlighting candidate genes for future functional exploration.

The observed temporal expression patterns suggest a phase-specific association of different *CsMYB* genes with the salt stress response. Our time-course expression analysis revealed a clear dichotomy in the response of CsMYB genes to salt stress. The rapid, transient upregulation of *CsMYB33* and *CsMYB44* as early as 12 h post-treatment coincides with the initial osmotic shock phase, suggesting their potential involvement in early stress signaling. This expression kinetics mirrors that of their putative orthologs, such as *AtMYB44* in *Arabidopsis*, which has been implicated in modulating early stress responses [9,29]. Conversely, the sustained upregulation of *CsMYB14*, *CsMYB78*, and *CsMYB79* from 24 h through to 5 days post-imbibition points to their possible contribution to longer-term adaptive processes. Such sustained expression profiles are characteristic of transcriptional regulators that may assist in maintaining cellular homeostasis under prolonged stress [30,31]. The parallel with the function of *TaMYB33* in wheat [10,32], which confers prolonged salt tolerance, underscores the potential evolutionary conservation of specific MYB TFs in enduring stress defense during early plant development. Promoter cis-element analysis provides crucial mechanistic insights.

In silico promoter analysis further implicates potential regulatory pathways linked to the observed expression dynamics. The significant enrichment of Abscisic Acid (ABA)-Responsive Elements (ABREs) in the promoters of key *CsMYBs*, such as *CsMYB14/78/79*, aligns their predicted regulatory activity with the core ABA signaling pathway—a central regulator of seed germination and abiotic stress responses [22,33,34]. This observation is consistent with the established ABA-MYB regulatory module documented in model species [8,15]. Concurrently, the abundance of Methyl Jasmonate (MeJA)-responsive elements (CGTCA/TGACG motifs) hints at a potentially prominent role for jasmonate signaling in *C. sativa*’s stress adaptation. Given the documented role of MYB TFs and JA signaling in regulating plant secondary metabolism [20,35], this finding invites the hypothesis of a possible link between early stress responses during germination and the later synthesis of specialized metabolites in mature plants—a connection that warrants future investigation.

A predicted protein–protein interaction network offers a systems-level perspective, though it remains to be experimentally validated. To generate hypotheses regarding the functional context of CsMYB proteins, a protein–protein interaction (PPI) network was computationally inferred based on orthology to experimentally characterized interactors of *A. thaliana* MYB proteins. It is critical to emphasize that this network is derived from orthology transfer and lacks direct experimental validation in *C. sativa*; Therefore, it should be interpreted primarily as a source of testable hypotheses. Nevertheless, the predictions are biologically coherent. For instance, the central positioning of sustained responders like *CsMYB14*, *CsMYB78*, and *CsMYB79* within the network, coupled with their predicted associations with core ABA signaling components (e.g., PP2C phosphatases, SnRK2 kinases) and ROS-scavenging enzymes (e.g., peroxidases, catalases), provides a plausible mechanistic framework for their role in long-term tolerance. Future validation through yeast two-hybrid, co-immunoprecipitation, or similar assays in *C. sativa* will be essential to confirm these interactions.

The downregulation of specific CsMYB genes may reflect an additional layer of transcriptional reprogramming under stress. The significant downregulation of *CsMYB58* and *CsMYB110* under salt stress reveals another strategic layer of transcriptional reprogramming. This suppression may represent a resource reallocation strategy, whereby genes promoting standard metabolic processes under favorable conditions are modulated to prioritize stress defense mechanisms. Similar repression of specific *MYB* members under stress has been documented in other species [7,20], indicating a potential adaptive principle, although the specific genes involved appear to be lineage-specific.

In conclusion, our study systematically characterizes the *CsMYB* family and proposes a coordinated, multi-phase regulatory framework for salt stress response during *C. sativa* seed germination based on correlative expression patterns of selected genes. The early, transient activity of *CsMYB33* and *CsMYB44* correlates with the initial osmotic phase, while the sustained expression of *CsMYB14*, *CsMYB78*, and *CsMYB79* is associated with the prolonged adaptation phase, potentially through ABA-mediated pathways and ROS management as suggested by in silico evidence. This model, supported by phylogenetic, structural, promoter, and expression data, establishes a robust framework for future functional genomics. The logical next steps involve functional validation through overexpression and knockout studies in *C. sativa* to confirm the roles of these candidate genes, followed by the identification of their direct target genes during germination. Ultimately, this work provides crucial genetic resources and candidate targets for molecular breeding aimed at enhancing salt tolerance, thereby supporting the development of resilient *C. sativa* varieties capable of stable establishment in saline-affected marginal environments.

## 4. Materials and Methods

### 4.1. Plant Materials and Seed Germination Conditions

Seeds of *C. sativa* L., Qingdama 5 (provided by the Daqing branch of Heilongjiang Academy of Agriculture Science, Daqing, China) were surface-sterilized with 2% (*v*/*v*) sodium hypochlorite solution (prepared from a stock solution of analytical grade, Sigma-Aldrich, St. Louis, MO, USA) for 10 min and subsequently rinsed five times with sterile distilled water. The sterilized seeds were then placed on sterile Whatman No. 1 filter paper (Cytiva, Marlborough, MA, USA) in 9 cm Petri dishes (30 seeds per dish), moistened with 10 mL of either sterile distilled water (control) or NaCl (analytical grade, Sinopharm Chemical Reagent Co., Ltd., Shanghai, China) solution for stress treatment. Germination was carried out in a growth chamber at 25 °C in complete darkness.

Preliminary experiments were conducted to determine the appropriate salt stress concentration by applying a gradient of NaCl solutions (0, 50, 100, 150, 200, and 500 mM). Based on the germination parameters, including mean germination time (MGT), germination index (GI), and time to 50% germination (T50), a concentration of 150 mM NaCl was selected for subsequent transcriptomic and qRT-PCR analyses. For the time-course expression analysis, seeds were germinated under 150 mM NaCl and sampled at critical stages of germination: 0 h (dry seed, post-imbibition start), 12 h (early imbibition phase), 24 h (radicle emergence phase for most seeds under control conditions), 48 h (early seedling growth), and 5 days post-imbibition (dpi) (seedling establishment). Control (water-treated) samples were collected at identical time points (0, 12, 24, 48 h, and 5 d) for direct comparison. Each time point consisted of three biological replicates (30 seeds per replicate). The collected samples were immediately frozen in liquid nitrogen and stored at −80 °C until RNA extraction.

### 4.2. Identification of MYB Genes in C. sativa

The identification of MYB transcription factors in the *C. sativa* genome was performed using a comprehensive bioinformatics pipeline. The genomic data, including the whole-genome sequence, protein sequences, and the corresponding annotation file (GFF3), were retrieved from the NCBI database (Assembly: ASM2167462v1).

To ensure a robust and complete identification, a dual strategy was employed [18,19]. First, a Hidden Markov Model (HMM) search was conducted using the HMM profile of the MYB DNA-binding domain (PF00249) obtained from the Pfam database. The HMMER 3.3.2 software suite was used to scan the entire *C. sativa* proteome with stringent cutoffs (E-value ≤ 1 × 10^−5^, score > 90). Second, a local BLASTP search was performed using a curated set of known *Arabidopsis thaliana* MYB protein sequences as queries against the *C. sativa* proteome, with an E-value threshold of 1 × 10^−5^.

The candidate sequences obtained from both approaches were merged, and duplicates were removed to create a non-redundant candidate list. To eliminate false positives, all candidate protein sequences were further verified for the presence of the canonical MYB domain using the SMART (Simple Modular Architecture Research Tool) database and the Conserved Domain Database (CDD) at NCBI. Only proteins containing a confirmed MYB domain were retained for subsequent analysis.

### 4.3. Phylogenetic, Motif, and Gene Structure Analysis

Multiple sequence alignment of the full-length CsMYB protein sequences was performed using the MUSCLE algorithm with default parameters. A phylogenetic tree was constructed using the Maximum Likelihood method in MEGA 12 software, with 1000 bootstrap replicates to assess the reliability of the tree nodes [19,20].

The conserved motifs within the CsMYB proteins were identified using the MEME Version 5.5.9 (Multiple Em for Motif Elicitation) suite, with the following parameters: maximum number of motifs set to 10, and the optimum motif width set to between 6 and 50 amino acids. The gene structure (exon-intron organization) was visualized based on the genomic DNA and CDS sequences of each *CsMYB* gene using the Gene Structure View function in TBtools. The phylogenetic tree, motif compositions, and gene structures were integrated and visualized using TBtools to illustrate their correlations.

### 4.4. Promoter Cis-Element Analysis

The 2000 bp genomic sequences upstream of the transcription start site (TSS) for each *CsMYB* gene were defined as promoter regions and extracted from the genome assembly file using TBtools. These promoter sequences were subsequently submitted to the PlantCARE database for a systematic in silico analysis of putative cis-acting regulatory elements. The analysis focused on identifying elements related to stress responses (e.g., MBS for drought, TC-rich repeats for defense) and hormone signaling (e.g., ABRE for ABA, CGTCA-motif for MeJA). The results were filtered, and the key elements were summarized and visualized as a heatmap using TBtools [21,22].

### 4.5. Synteny Analysis

To investigate the evolutionary history of the *CsMYB* gene family, intra-genomic and inter-genomic synteny analyses were performed. The genomic data for *A. thaliana* (TAIR10), *O. sativa* (IRGSP-1.0), and Z. *mays* (RefGen_v4) were downloaded from EnsemblPlants. The MCScanX algorithm embedded in TBtools was used to detect syntenic blocks with a BLASTP (https://blast.ncbi.nlm.nih.gov/) E-value cutoff of 1 × 10^−10^. The synteny relationships were visualized using the Advanced Circos function in TBtools.

### 4.6. Expression Analysis of Salt Stress Related CsMYBs During Seed Germination

#### 4.6.1. RNA-Sequencing and Candidate Gene Selection

To identify candidate *CsMYB* genes responsive to salt stress, a preliminary RNA-seq experiment was conducted as a screening approach at a single critical time point (24 h post-imbibition, hpi). Total RNA was extracted from seeds germinated under 150 mM NaCl at 24 hpi using the TRIzol^®^ reagent (Invitrogen, Carlsbad, CA, USA) following the manufacturer’s protocol with three independent biological replicates. RNA integrity was assessed using an Agilent 2100 Bioanalyzer (Agilent Technologies, Santa Clara, CA, USA). Libraries were constructed with the NEBNext^®^ Ultra™ II RNA Library Prep Kit (New England Biolabs, Ipswich, MA, USA) and sequenced on an Illumina NovaSeq 6000 platform (Illumina, San Diego, CA, USA) in 150 bp paired-end mode, generating approximately 40 million reads per sample. Raw reads were quality-trimmed using Trimmomatic v0.39 and aligned to the *C. sativa* reference genome (Assembly: GCF_900626175.2; https://www.ncbi.nlm.nih.gov/assembly/GCF_900626175.2, accessed on 14 February 2019). with HISAT2 v2.2.1. Transcript assembly and quantification were performed using StringTie v2.1.4, and differential expression analysis was conducted with DESeq2 v1.34.0. Genes with |log_2_FoldChange| > 1 and adjusted *p*-value < 0.05 were considered differentially expressed [12,25]. From the 115 identified *CsMYB* genes, 10 candidates were selected based on the following criteria: (1) significant differential expression in the 24 hpi RNA-seq dataset under salt stress; (2) presence of stress- or hormone-related cis-elements in their promoters; and (3) phylogenetic proximity to known stress-responsive MYB orthologs in *Arabidopsis thaliana*. The selected genes were *CsMYB14*, *CsMYB19*, *CsMYB33*, *CsMYB44*, *CsMYB58*, *CsMYB63*, *CsMYB78*, *CsMYB79*, *CsMYB101*, *and CsMYB110*.

#### 4.6.2. Quantitative Real-Time PCR (qRT-PCR) Validation

Total RNA was extracted from frozen seed/seedling samples (including both NaCl-treated and matched water control samples from all time points) using the Plant Total RNA Extraction Kit (Tiangen Biotech, Beijing, China) according to the manufacturer’s instructions. Genomic *DNA* was removed with DNase I (RNase-free, Thermo Fisher Scientific, Waltham, MA, USA). RNA integrity was checked by 1% agarose gel electrophoresis, and concentration was measured with a NanoDrop™ One spectrophotometer (Thermo Fisher Scientific). First-strand cDNA was synthesized from 1 µg of total RNA using the PrimeScript™ RT Reagent Kit with gDNA Eraser (Takara Bio, Kusatsu, Japan). Gene-specific primers for the 10 selected *CsMYB* genes were designed with Primer Premier 5.0 (Premier Biosoft, Palo Alto, CA, USA); sequences are listed in Supplementary Table S1. Primer efficiency for each pair was validated using a standard curve from a cDNA dilution series, and only primers with efficiencies between 90% and 110% and a single peak in melt-curve analysis were used. The linear dynamic range of amplification was confirmed. The reference gene *CsActin* was selected based on its stable expression across all treatments and time points in our experimental system (validation data provided in Supplementary Materials). qRT-PCR was performed in triplicate for each biological replicate using the SYBR Green Premix Pro Taq HS qPCR Kit (Accurate Biology, Changsha, China) on a CFX96 Touch™ Real-Time PCR Detection System (Bio-Rad Laboratories, Hercules, CA, USA). The thermal cycling protocol was: 95 °C for 30 s, followed by 40 cycles of 95 °C for 5 s and 60 °C for 30 s. A melt-curve analysis was added to verify amplification specificity. The expression levels of target genes were normalized to the internal reference gene *CsActin*, which exhibited stable expression under our experimental conditions. Relative expression was calculated using the 2^−ΔΔCT^ method [36]. For each time point, expression in salt-treated samples was compared to that in the corresponding water-treated controls to distinguish salt-specific responses from developmental expression patterns.

### 4.7. Protein–Protein Interaction Network Prediction

To generate testable hypotheses regarding potential functional partnerships, a protein–protein interaction (PPI) network for the CsMYB proteins was predicted in silico using the STRING database (version 12.0) [27]. The protein sequences of the key *CsMYBs* were used as queries, and the network was built based on known and predicted interactions from the model plant *Arabidopsis thaliana* (orthology transfer). A high confidence score (≥0.7) was set as the cutoff. It should be noted that this is a predictive analysis based on orthology, and the expression data generated in this study were not integrated into the network construction. Experimental validation in *C. sativa* and integration with co-expression data remain important future directions. The resulting interaction network was visualized using Cytoscape software.

### 4.8. Statistical Analsysis

All experiments were conducted with at least three biological replicates. Data from qRT-PCR and germination assays are presented as mean ± standard deviation (SD). Statistical analysis was performed using OriginPro 2018b (OriginLab Corporation, Northampton, MA, USA). For multiple comparisons, one-way analysis of variance (ANOVA) followed by the Least Significant Difference (LSD) post hoc test was used. A probability value of *p* < 0.05 was considered statistically significant.

## 5. Conclusions

In conclusion, our findings delineate a temporal and functional specialization within the *CsMYB* family during salt-stressed seed germination. Early responders like *CsMYB33/44* likely manage the initial osmotic shock, while sustained expressers like *CsMYB14/78/79* are crucial for enduring tolerance, possibly through ABA-dependent pathways and ROS management. The suppression of certain *CsMYBs* further illustrates the metabolic trade-offs inherent to stress adaptation. This model provides a robust framework for future functional studies. Validating the roles of these candidate *CsMYBs* through transgenic approaches and identifying their direct target genes during germination will be critical steps towards unlocking their potential for breeding salt-tolerant *C. sativa* varieties, ensuring stable germination and seedling establishment in saline-affected soils.

## Data Availability

The original contributions presented in this study are included in the article and Supplementary Materials. Further inquiries can be directed to the corresponding authors.

## Supplementary Materials

The following supporting information can be downloaded at: https://www.mdpi.com/article/10.3390/ijms27021087/s1.

## References

[1] Zhu J.K. (2016). Abiotic stress signaling and responses in plants. Cell.

[2] Gupta A., Rico-Medina A., Caño-Delgado A.I. (2020). The physiology of plant responses to drought. Science.

[3] Yang Y., Guo Y. (2018). Elucidating the molecular mechanisms mediating plant salt-stress responses. New Phytol..

[4] Kissoudis C., van de Wiel C., Visser R.G.F., van der Linden G. (2016). Future-proof crops for multiple abiotic stresses: The case of drought and heat. Theor. Appl. Genet..

[5] Huang G.T., Ma S.L., Bai L.P., Ma H., Jia P., Liu J., Zhong M. (2021). Signal transduction during cold, salt, and drought stresses in plants. Mol. Plant.

[6] Dubos C., Stracke R., Grotewold E., Weisshaar B., Martin C., Lepiniec L. (2010). MYB transcription factors in Arabidopsis. Trends Plant Sci..

[7] Ding P., Tang P., Li X., Haroon A., Nasreen S., Noor H., Attia K.A., Abushady A.M., Wang R., Cui K. (2024). Genome-wide identification, phylogeny and expression analysis of the R2R3-MYB gene family in quinoa (*Chenopodium quinoa*) under abiotic stress. Funct. Plant Biol..

[8] Xiong H., Li J., Liu P., Duan J., Zhao Y., Guo X., Li Y., Zhang H., Ali J., Li Z. (2014). Overexpression of OsMYB48-1, a Novel MYB-Related Transcription Factor, Enhances Drought and Salinity Tolerance in Rice. PLoS ONE.

[9] Baloglu M.C., Inal B., Kavas M., Unver T. (2014). Diverse expression pattern of wheat transcription factors against abiotic stresses in wheat species. Gene.

[10] Wang R.K., Cao Z.H., Hao Y.J. (2013). Overexpression of a R2R3 MYB gene MdSIMYB1 increases tolerance to multiple stresses in transgenic tobacco and apples. Physiol. Plant..

[11] Wei Q., Zhang F., Sun F., Luo Q., Wang R., Hu R., Chen M., Chang J., Yang G., He G. (2017). A wheat MYB transcriptional repressor TaMyb1D regulates phenylpropanoid metabolism and enhances tolerance to drought and oxidative stresses in transgenic tobacco plants. Plant Sci..

[12] Cheng X., Deng G., Su Y., Liu J., Yang Y., Du G.H., Chen Z.Y., Liu F.H. (2016). Protein mechanisms in response to NaCl-stress of salt-tolerant and salt-sensitive industrial hemp based on iTRAQ technology. Ind. Crops Prod..

[13] Laverty K.U., Stout J.M., Sullivan M.J., Shah H., Gill N., Holbrook L., Page J.E. (2019). A physical and genetic map of *Cannabis sativa* identifies extensive rearrangements at the THC/CBD acid synthase loci. Genome Res..

[14] Jiang Z., Wang D., Che Y., Amanullah S., Zhang L., Jie S., Yang W., Wang M., Wang L., Qi G. (2025). Transcriptomic sequencing and expression verification of identified genes modulating the alkali stress tolerance and endogenous photosynthetic activities of industrial hemp plant. PLoS ONE.

[15] Zhang D.H., Wang X., Zhou C., Chen Y., Wang X., Wang S., He S., Guo Y., Liu Z., Chen M. (2024). Transcription factor DIVARI-CATA1 positively modulates seed germination in response to salinity stress. Plant Physiol..

[16] Cao K., Sun Y., Zhang X., Zhao Y., Bian J., Zhu H., Wang P., Gao B., Sun X., Hu M. (2023). The miRNA–mRNA regulatory networks of the response to NaHCO_3_ stress in industrial hemp (*Cannabis sativa* L.). BMC Plant Biol..

[17] Ambawat S., Sharma P., Yadav N.R., Yadav R.C. (2013). MYB transcription factor genes as regulators for plant responses: An overview. Physiol. Mol. Biol. Plants.

[18] Katiyar A., Smita S., Lenka S.K., Rajwanshi R., Chinnusamy V., Bansal K.C. (2012). Genome-wide classification and expression analysis of MYB transcription factor families in rice and Arabidopsis. BMC Genom..

[19] Chen Y., Yang X., He K., Liu M., Li J., Gao Z., Lin Z., Zhang Y., Wang X., Qiu X. (2006). The MYB transcription factor superfamily of Arabidopsis: Expression analysis and phylogenetic comparison with the rice MYB family. Plant Mol. Biol..

[20] Liu J., Osbourn A., Ma P. (2015). MYB Transcription Factors as Regulators of Phenylpropanoid Metabolism in Plants. Mol. Plant.

[21] Roy S. (2016). Function of MYB domain transcription factors in abiotic stress and epigenetic control of stress response in plant genome. Plant Signal. Behav..

[22] Seo P.J., Xiang F., Qiao M., Park J.Y., Lee Y.N., Kim S.G., Park C.M. (2009). The MYB96 transcription factor mediates abscisic acid signaling during drought stress response in Arabidopsis. Plant Physiol..

[23] Cui R., An X. (2024). Research progress of MYB transcription factor family in plant stress resistance. Not. Bot. Horti Agrobot. Cluj-Napoca.

[24] Wu X., Xia M., Su P., Zhang Y., Tu L., Zhao H., Gao W., Huang L., Hu Y. (2024). MYB transcription factors in plants: A comprehensive review of their discovery, structure, classification, functional diversity and regulatory mechanism. Int. J. Biol. Macromol..

[25] He Y., Li W., Lv J., Jia Y., Wang M., Xia G. (2012). Ectopic expression of a wheat MYB transcription factor gene, TaMYB73, improves salinity stress tolerance in *Arabidopsis thaliana*. J. Exp. Bot..

[26] Oh J.E., Kwon Y., Kim J.H., Noh H., Hong S.W., Lee H. (2011). A dual role of MYB60 in stomatal regulation and root growth of *Arabidopsis thaliana* under drought stress. Plant Mol. Biol..

[27] Miao Y., Laun T., Zimmermann P., Zentgraf U. (2004). Targets of the WRKY53 transcription factor and its role during leaf senescence in Arabidopsis. Plant Mol. Biol..

[28] Berni R., Thiry M., Hausman J.F., Lutts S., Guerriero G. (2024). Eustress and Plants: A Synthesis with Prospects for *Cannabis sativa* Cultivation. Horticulturae.

[29] Mao X., Jia D., Li A., Zhang H., Tian S., Zhang X., Jing R. (2011). Transgenic expression of TaMYB2A confers enhanced tolerance to multiple abiotic stresses in Arabidopsis. Funct. Integr. Genom..

[30] Wang Y., Liu W., Jiang H., Mao Z., Wang N., Jiang S., Xu H., Yang G., Zhang Z., Chen X. (2019). The R2R3-MYB transcription factor MdMYB24-like is involved in methyl jasmonate-induced anthocyanin biosynthesis in apple. Plant Physiol. Biochem..

[31] Agarwal P.K., Agarwal P., Reddy M.K., Sopory S.K. (2006). Role of DREB transcription factors in abiotic and biotic stress tolerance in plants. Plant Cell Rep..

[32] Liao Y., Zou H.F., Wang H.W., Zhang W.K., Ma B., Zhang J.S., Chen S.Y. (2008). Soybean GmMYB76, GmMYB92, and GmMYB177 genes confer stress tolerance in transgenic Arabidopsis plants. Cell Res..

[33] Vannini C., Locatelli F., Bracale M., Magnani E., Marsoni M., Osnato M., Coraggio I. (2004). Overexpression of the rice Osmyb4 gene increases chilling and freezing tolerance of *Arabidopsis thaliana* plants. Plant J..

[34] Li C., Ng C.K.Y., Fan L.M. (2015). MYB transcription factors, active players in abiotic stress signaling. Environ. Exp. Bot..

[35] Chen L., Song Y., Li S., Zhang L., Zou C., Yu D. (2012). The role of WRKY transcription factors in plant abiotic stresses. Biochim. Biophys. Acta.

[36] Livak K.J., Schmittgen T.D. (2001). Analysis of relative gene expression data using real-time quantitative PCR and the 2^−ΔΔCT^ method. Methods.

