# Supplementary material for: Genome-Wide Identification, Phylogenetic Analysis and Salt-Responsive Expression Profiling of the MYB Transcription Factor Family in Cannabis sativa L. During Seed Germination
Source: Int J Mol Sci. 2026 Jan 22;27(2):1087. doi: 10.3390/ijms27021087 (PMC12841950; doi:10.3390/ijms27021087)
Supplement: Supplementary file 1 [file ijms-27-01087-s001.zip › Supplementary Table S1. Gene-specific primers used for qRT-PCR analysis.pdf]

Supplementary Table S1. Gene-specific primers used for qRT-PCR analysis

Gene Name	Primer (5′—3′)	Sequence (5′—3′)	Amplicon Size (bp)
CsMYB14	Forward	ATGGCGAGAAAGAGGAGAACC	152
	Reverse	TCCTTGGTGGTGGTGTAGGT	
CsMYB19	Forward	CCAAGCTCAACAACCACAACG	138
	Reverse	GGTGGTGTTGTAGGCGTTGT	
CsMYB33	Forward	GGAGGAGGAAGAGGAGGAGAA	141
	Reverse	CCTTGTTGTTGTGGTGGTGT	
CsMYB44	Forward	AAGGAAGCGGAAGAGGACAA	145
	Reverse	TGGTGGTGGTTGTAGGTGTT	
CsMYB58	Forward	CCAACAACCACCACCACAGA	128
	Reverse	GGTTGTGGTGGTTGTGGTAG	
CsMYB63	Forward	AAGGAGAAGGAGCGGAAGAA	135
	Reverse	CCTTGGTGGTTGTGGTAGGT	
CsMYB78	Forward	GGAGGAAGAGGAGCGGAAGA	148
	Reverse	TGGTGGTGGTAGGTGGTTGT	
CsMYB79	Forward	AAGGAAGCGGAAGAGGAGGA	129
	Reverse	GGTGGTGGTAGGTGGTTGTT	
CsMYB101	Forward	CCAAGCTCAACAACCACAAC	135
	Reverse	GGTGGTGTTGTAGGCGTTGA	
CsMYB110	Forward	GGAGGAGGAAGAGGAGGAGA	120
	Reverse	CCTTGGTGGTGGTGTAGGC	
CsActin	Forward	GTGACAATGGAACTGGAATGG	115
	Reverse	TCCTCCAATCCAAACACTGTC	
